# Investigation of Outbreaks of *Salmonella enterica* Serovar Typhimurium and Its Monophasic Variants Using Whole-Genome Sequencing, Denmark

**DOI:** 10.3201/eid2310.161248

**Published:** 2017-10

**Authors:** Pernille Gymoese, Gitte Sørensen, Eva Litrup, John Elmerdal Olsen, Eva Møller Nielsen, Mia Torpdahl

**Affiliations:** Statens Serum Institut, Copenhagen, Denmark (P. Gymoese, G. Sørensen, E. Litrup, E.M. Nielsen, M. Torpdahl);; Technical University of Denmark, Lyngby, Denmark (G. Sørensen);; University of Copenhagen, Frederiksberg, Denmark (J.E. Olsen)

**Keywords:** Salmonella Typhimurium, outbreaks, surveillance, genomics, sequence analysis, molecular typing, single-nucleotide polymorphism, SNP, foodborne disease, bacteria, food safety, whole-genome sequencing, monophasic variants, multilocus variable-number tandem-repeat analysis, MLVA, Denmark

## Abstract

Whole-genome sequencing is rapidly replacing current molecular typing methods for surveillance purposes. Our study evaluates core-genome single-nucleotide polymorphism analysis for outbreak detection and linking of sources of *Salmonella enterica* serovar Typhimurium and its monophasic variants during a 7-month surveillance period in Denmark. We reanalyzed and defined 8 previously characterized outbreaks from the phylogenetic relatedness of the isolates, epidemiologic data, and food traceback investigations. All outbreaks were identified, and we were able to exclude unrelated and include additional related human cases. We were furthermore able to link possible food and veterinary sources to the outbreaks. Isolates clustered according to sequence types (STs) 19, 34, and 36. Our study shows that core-genome single-nucleotide polymorphism analysis is suitable for surveillance and outbreak investigation for *Salmonella* Typhimurium (ST19 and ST36), but whole genome–wide analysis may be required for the tight genetic clone of monophasic variants (ST34).

The foodborne pathogen *Salmonella* is responsible for tens of millions of human infections worldwide each year (*1*). It constitutes a substantial health and economic burden, especially in developing countries (*1*). Fast, accurate, and highly discriminatory typing methods are crucial for detecting outbreaks, identifying sources of the outbreaks, and preventing further spread of the bacteria as part of effective surveillance.

In Denmark, 1,122 cases of human *Salmonella* infections were registered in 2014. *Salmonella enterica* serovar Typhimurium accounted for 17.6% of cases, and its monophasic variants accounted for 20.5%. Cases are often associated with consumption of swine and poultry products (*2*). In Denmark, as in many countries worldwide, the monophasic *Salmonella* Typhimurium variants have emerged in the past decades (*3*–*8*). The monophasic variants are circulating in multiple clonal lineages, and owing to the relatively rapid emergence of the clones that often also exhibit multidrug resistance, these types of monophasic variants are considered an important epidemic health risk (*3*,*9*–*11*).

Whole-genome sequencing (WGS) is a widely used technique for molecular subtyping of bacteria, and it is replacing the more laborious current molecular typing methods. The vast amount of data provided by this method not only enables high-resolution typing for surveillance but also provides valuable additional data regarding further characterization of emerging clones based on genetic differences and evolutionary studies. Several studies have proven WGS-based typing to have an enhanced discriminatory power in comparison to current molecular typing methods used for *Salmonella* (*12*–*19*), although few studies have evaluated WGS analysis in real-time surveillance. A retrospective study of *Salmonella* Enteritidis showed that single-nucleotide polymorphism (SNP)–based WGS analysis was suitable for surveillance purposes (*15*). However, the authors also emphasized the importance of evaluation and interpretation of the SNP-based analysis within serovars or even lineages before applying the method in real-time surveillance.

In Denmark, surveillance of *Salmonella* Typhimurium and its monophasic variants is conducted at Statens Serum Institut (human clinical isolates) and the National Food Institute (food and veterinary isolates). Surveillance is based on serotyping, drug-susceptibility testing, and multilocus variable-number tandem-repeat analysis (MLVA). The aim of our study was to evaluate WGS as a typing method for routine surveillance of *Salmonella* Typhimurium and its monophasic variants. To do so, we selected an already typed collection of strains from 2013 and 2014 and reanalyzed them to redefine outbreaks and detect outbreak sources based on core-genome SNP analysis.

## Material and Methods

We selected 372 isolates of *Salmonella* Typhimurium and its monophasic variants for this study (Technical Appendix 1). The collection included 292 human clinical isolates from the national surveillance system in Denmark (Statens Serum Institut, Copenhagen) collected during January 2013–April 2013 (previously sequenced isolates) and June 2014–October 2014 (isolates sequenced during this study). During the 7 months of surveillance, 8 outbreaks were previously defined based on epidemiologic data, serotyping, drug-susceptibility testing, and MLVA (Table 1). Outbreak investigations during that period were initiated when 5 isolates with an indistinguishable MLVA profile were collected within a 4-week period. The investigations included patient interviews, typing of food and veterinary isolates, and examination of isolates with closely related MLVA (1 locus difference) and resistance profiles.

**Table 1:** Previously defined outbreaks included in the data collection in an outbreak investigation of *Salmonella enterica* serovar Typhimurium and its monophasic variants, Denmark*

Outbreak	No. sequenced strains	Year	Outbreak characteristics	Serovar variant	Food/veterinary isolates linked to outbreak
A	6	2014	Same MLVA and resistance profile	Typhimurium	No
B	35	2013	Same MLVA, various resistance profiles	Typhimurium	Yes
C	7	2013	Same MLVA and resistance profile	Typhimurium	No
D	8	2013	Same MLVA, various resistance profiles	Typhimurium	No
E	24	2014	Same MLVA, various resistance profiles	Monophasic	Yes
F	19	2014	Various MLVA, same resistance profile	Monophasic	Yes
G	14	2014	Same MLVA, various resistance profiles	Monophasic	No
H	5	2014	Same MLVA and resistance profile	Monophasic	Yes

*MLVA, multilocus variable-number tandem-repeat analysis.

In addition, we selected 80 food and veterinary isolates linked or possibly linked to the outbreaks from the National Food Institute collection in Denmark (DTU Food, Technical University of Denmark, Lyngby, Denmark). The food and veterinary isolates were isolated from swine, poultry, cattle, and feed in 2010, 2013, and 2014.

### WGS and Sequence Analysis

We analyzed all human isolates by using WGS at Statens Serum Institut’s Department of Microbiology and Infection Control and sequenced food and veterinary isolates at the Technical University of Denmark’s National Food Institute. We sequenced isolates by using an Illumina Miseq (Illumina, San Diego, CA, USA). All sequences were de novo assembled and sequence type (ST) determined. We identified core-genome SNPs by using an in-house SNP pipeline, and we then analyzed a selected subgroup of isolates by using the SNP pipelines NASP (*20*) and CSI Phylogeny 1.2 (*21*). A description of sequencing procedures and sequence analysis is provided (Technical Appendix 2). We assessed quality of the sequences and excluded 6 isolates from the study because of poor quality. Additional information on sequences also is provided (Technical Appendix 1). Sequence reads were deposited in the European Nucleotide Archive (study accession no. PRJEB14853).

## Results

**Figure 1:**
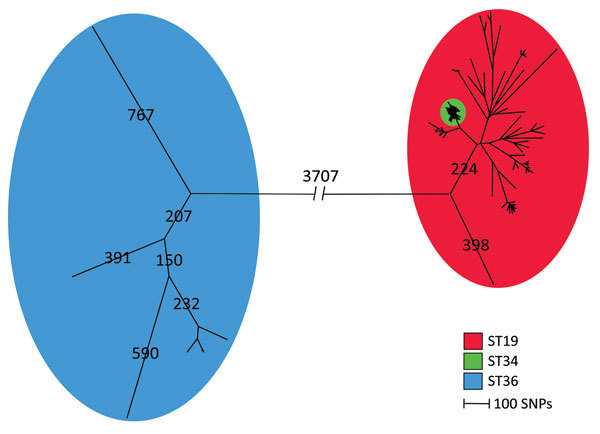
Maximum-parsimony tree of 288 human isolates and 78 linked food and veterinary isolates of *Salmonella enterica* serovar Typhimurium and its monophasic variants based on core-genome SNP analysis with the complete genome of *Salmonella* Typhimurium 14028S as the reference genome in an outbreak investigation of *Salmonella* Typhimurium and its monophasic variants, Denmark. Branches are labeled with number of SNP differences. Three ST groups are highlighted: ST19, ST34, and ST36. SNP, single-nucleotide polymorphism; ST, sequence type.

We detected core-genome SNPs in the entire isolate collection by using the complete genome of *Salmonella* Typhimurium 14028S (ST19) as the reference genome. The SNP analysis resulted in 14,326 SNPs. We constructed a maximum-parsimony tree from the core-genome SNPs and observed 3 ST-specific groups (Figure 1); 1 group mainly consisted of ST19 strains, 1 solely consisted of ST34 stains, and 1 solely consisted of ST36 strains. A long branch separated all 11 Typhimurium ST36 isolates from the remaining isolates with 3,707 SNPs. Furthermore, we observed a distinct cluster of 242 isolates of ST34. The close genetic cluster included isolates of both serovar Typhimurium and monophasic variants, with the monophasic variants being most prevalent. The remaining 113 isolates clustering together were ST19, ST376, ST568, and ST2212, all identified as serovar Typhimurium.

### Outbreak Investigation

We analyzed the 3 observed ST groups separately. Core-genome SNPs were detected by using an internal de novo assembled reference genome for each ST group. We examined the 8 previously defined outbreaks (outbreaks A–H; Table 1) on the basis of the genetic relatedness of the isolates, the epidemiologic data, and the food traceback investigations. We identified and redefined all 8 outbreaks on the basis of the SNP analysis (Table 2). We also plotted the distribution of the outbreaks over time (Technical Appendix 2, Figure).

**Table 2:** New definitions of previously defined outbreaks based on core-genome SNP analysis in an outbreak investigation of *Salmonella enterica* serovar Typhimurium and its monophasic variants, Denmark*

Outbreak	No. human cases	Included/excluded compared with previously defined	Food/veterinary isolates	Sources	No. SNPs	Maximum SNP distance	SNP distance from nearest neighbor
A	6	–	0	–	8	7	143
B	37	+2	2	Swine	11	4	34
C	7	–	0	–	0	0	64
D	7	+1/–2	4	Swine	2 (6)	2 (6)	3 (7)
E	20	+1/–5	13	Swine/cattle	6 (7)	3 (4)	3 (4)
F	22	+3	2	Cattle	4	3	20
G	9	−5	0	–	2	2	31
H	5	–	1	Swine	0	0	4 (7)

*Previous outbreaks shown in Table 1. SNPs in parentheses are derived from reanalysis of closely related clusters with an internal de novo assembled reference genome. SNP, single-nucleotide polymorphism.

### The ST36 Group

**Figure 2:**
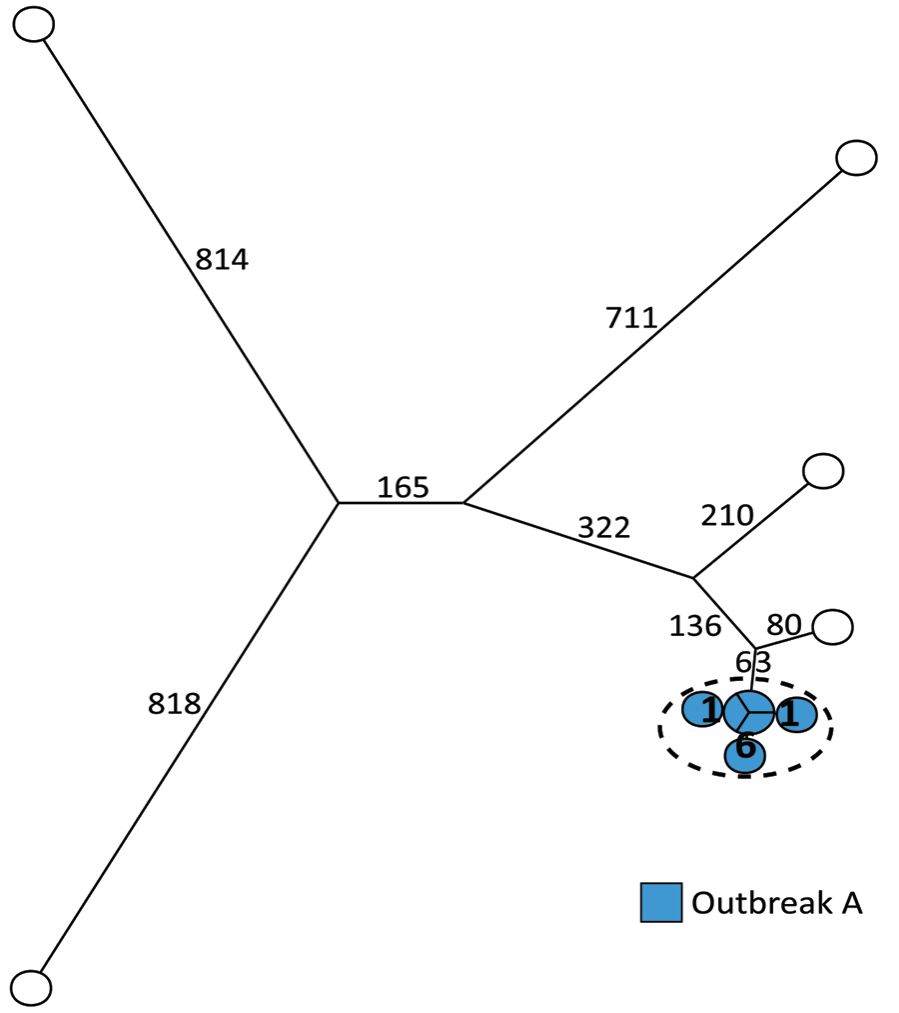
Maximum-parsimony tree of 11 human isolates of *Salmonella* *enterica* serovar Typhimurium ST36 based on core-genome SNP analysis with an internal de novo assembled ST36 genome as the reference genome in an outbreak investigation of *Salmonella* Typhimurium and its monophasic variants, Denmark. Branches are labeled with number of SNP differences. One outbreak (outbreak A) was included. Isolates highlighted in blue belong to outbreak A as previously defined by MLVA; isolates inside the dotted circle are outbreak isolates as defined by the SNP analysis. MLVA, multilocus variable-number tandem-repeat analysis; SNP, single-nucleotide polymorphism; ST, sequence type.

We analyzed 11 ST36 isolates and detected 3,146 core-genome SNPs with SNP distances between isolates ranging from 0 to 1,694. The previous definition of outbreak A included 6 human cases and no suspected food and veterinary isolates (Table 1). The SNP analysis clustered all 6 isolates with a SNP distance between the isolates of 0 to 7 SNPs. Nearest neighbor isolate was separated from the cluster with 143 SNPs, clearly differentiating the outbreak cluster from the remaining isolates (Figure 2; Table 2).

### The ST19 Group

**Figure 3:**
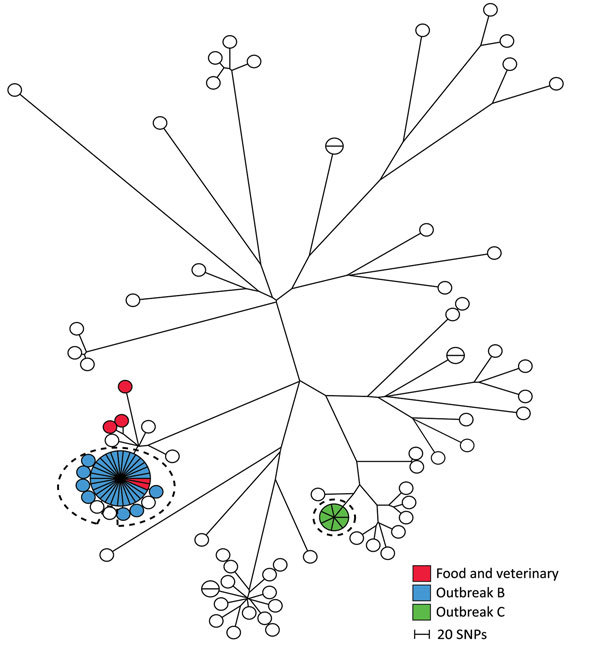
Maximum-parsimony tree of 98 human isolates and 5 linked food and veterinary isolates of *Salmonella* *enterica* serovar Typhimurium with mainly ST19 based on core-genome SNP analysis with an internal de novo assembled ST19 genome as the reference genome in an outbreak investigation of *Salmonella* Typhimurium and its monophasic variants, Denmark. Branch lengths correspond to number of SNPs. Isolates belonging to outbreaks B and C are as previously defined by MLVA. Isolates inside the dotted circles are outbreak isolates as defined by the SNP analysis. MLVA, multilocus variable-number of tandem-repeat analysis; SNP, single-nucleotide polymorphism; ST, sequence type.

The ST19 group comprised 98 human isolates and 5 food and veterinary isolates. Within the ST19 group, we detected 6,549 SNPs with distances between isolates ranging from 0 to 982 SNPs (Figure 3). Two outbreaks (B and C) were detected in 2013; B comprised 35 human isolates, and C comprised 7 human isolates (Table 1).

A tight genetic cluster with 0 to 4 SNP differences between the isolates comprised all 35 isolates previously defined in outbreak B. Two additional human isolates from 2013 with closely related MLVA profiles were located in this cluster and regarded as part of the outbreak, as defined by the SNP analysis. Likewise, 1 isolate from 2014 clustered with the outbreak cases but was not included in the new outbreak definition because of the difference in time. Patient interviews pointed to consumption of pork as the likely contamination source. Two suspected food isolates from pork with the same MLVA profile were, at the time, collected from 2 different meat-distributing companies. However, we could not confirm a clear connection between the food isolates and the human cases. Our SNP analysis showed that the 2 suspected isolates were located in the outbreak cluster and therefore provided additional evidence that pork was the likely source of the outbreak. The cluster was separated from the nearest neighbor isolate with 34 SNPs (Figure 3; Table 2).

All 7 isolates previously defined in outbreak C had identical core-genome SNPs. No food or veterinary isolates were linked to the cases, and the outbreak cluster was separated from the nearest neighbor with 64 SNPs (Figure 3; Table 2).

### The ST34 Group

Most of the isolates observed in this study were ST34, and this ST group was dominated by the monophasic variants. The ST34 group included 169 human isolates and 73 possibly linked food and veterinary isolates, and 5 outbreaks were detected (outbreaks D–H; Table 1).

**Figure 4:**
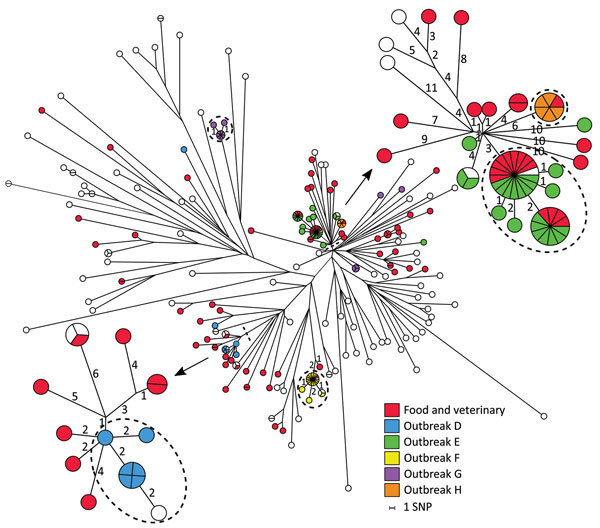
Maximum-parsimony tree of 169 human isolates and 73 linked food and veterinary isolates of *Salmonella* *enterica* serovar Typhimurium and the monophasic variants ST34 based on core-genome SNP analysis with an internal de novo assembled ST34 genome as the reference genome in an outbreak investigation of *Salmonella* Typhimurium and its monophasic variants, Denmark. Some branches are labeled with the number of SNP differences, and branch lengths correspond to the number of SNPs. Isolates belonging to outbreaks D, E, F, G, and H are as previously defined by MLVA. Isolates inside the dotted circles are outbreak isolates as defined by the SNP analysis. Two selected subgroups were reanalyzed separately with internal de novo assembled reference genomes (arrows). MLVA, multilocus variable-number tandem-repeat analysis; SNP, single-nucleotide polymorphism; ST, sequence type.

The SNP analysis of the 242 isolates resulted in 1,488 core-genome SNPs. SNP distances ranged from 0 to 95. Based on core-genome SNPs, the genetic relation between isolates was distinctly more close in comparison to ST36 and ST19. For some clusters, few SNPs separated the isolates, so defining outbreaks based on the analysis was complicated. We recalculated 2 close clusters, which included outbreaks D, E, and H, separately with an internal de novo assembled reference genome to obtain a higher resolution (Figure 4; Table 2). The recalculation added a few extra SNPs; however, conclusions were still not clear cut. Analyzing statistics from our SNP pipeline revealed that ≈20% of the reference genome was discarded when all sequences were analyzed using the closed reference genome (Table 3). Likewise, in some cases, 10% of the reference genome was not used when analyzing an apparently closely related cluster separately. To rule out whether the disregarded data were attributable to the SNP pipeline used, we additionally analyzed the cluster including outbreaks E and H by using the 2 alternative core-genome SNP pipelines NASP (*20*) and CSI Phylogeny 1.2 (*21*). From our in-house pipeline, we identified 374 core-genome SNPs within this cluster. The NASP pipeline identified 404 SNPs, and CSI Phylogeny identified 361 SNPs. No further obvious changes were observed in the overall phylogeny in this cluster or for the resolution within the outbreaks, supporting the robustness of our pipeline.

**Table 3:** Statistical data from core-genome SNP analysis of different subgroups with complete genome of *Salmonella enterica* serovar Typhimurium 14028S or an internal de novo assembled genome as reference in an outbreak investigation of *Salmonella* Typhimurium and its monophasic variants, Denmark*

Isolates within	Selection of isolates	Size of core-genome used, bp			% Reference genome used			Called SNPs	
		14028S	Internal de novo		14028S	Internal de novo		14028S	Internal de novo
All	All	3806685	–		78.16	–		14,326 (10,163)	–
ST36	All	4527849	4494995		92.97	95.32		2,887	3,146
	Outbreak A	4604894	4611321		94.55	97.78		8	8
ST19	All	4314989	4304908		88.60	90.34		6,596	6,549 (6,004)
	Outbreak B	4648245	4662681		95.44	97.85		16	16
	Outbreak C	4711565	4720046		96.74	99.02		0	0
ST34	All	4055543	4034423		83.27	81.55		1,490	1,488 (1,091)
	Outbreak D	4665201	4771546		95.79	97.39		32	34
	Outbreak E	4706675	4871858		96.64	98.48		7	7
	Outbreak F	4705075	4836637		96.61	98.41		9	10
	Outbreak G	4694550	4826771		96.39	98.00		2	3
	Outbreak H	4734518	4886190		97.21	98.84		0	0

*SNPs in parentheses are called SNPs with inclusion of poor-quality sequences. SNP, single-nucleotide polymorphism; ST, sequence type.

Outbreak D, detected in 2013, previously comprised 8 human isolates. From our analysis, we could include 1 additional human case and exclude 2, on the basis of the genetic relatedness of the isolates (Figure 4). Patient interviews revealed a likely source being consumption of pork from a specific butcher. No relevant food samples from the butcher, patient households, or the companies distributing meat to the butcher were available. Isolates with the same MLVA profile were, at the time, isolated from different slaughterhouses. The SNP analysis linked 3 swine isolates collected from 3 different slaughterhouses with 2–4 SNP differences with the human cases. Another food isolate from pork separated with 6 SNPs was also likely connected to the outbreak. Four food and veterinary isolates, also separated with few SNPs to the outbreak cases, were collected in 2014 and therefore not considered as part of the outbreak. The SNP analysis provided additional evidence for the connection with consumption of pork, further indicating multiple possible sources and the presence of the strain in the food production in 2014.

A large outbreak (outbreak E) was detected in 2014; our collection included 24 isolates from that outbreak. Of the 24 isolates, 23 clustered with few SNP differences (Figure 4). Because the outbreak isolates were located in a closely related cluster in the ST34 group, a clear outbreak definition based on SNP analysis was difficult to make. One human isolate was clearly separated from the cluster and could be excluded as an outbreak case. One additional isolate with a different MLVA profile clustered with 0 SNPs to cases and was included. The probable source of the outbreak was connected to consumption of pork. Samples from swine with the same MLVA profile were collected from a slaughterhouse and suspected as the primary source. Traceback investigations pointed to a specific swine herd with high *Salmonella* carriage rate supplying swine to the slaughterhouse during the same period. We collected additional samples from a company cutting and distributing meat from the slaughterhouse and from products from companies receiving meat from the primary sources. Isolates from the suspected primary and secondary sources clustered with 0 SNPs to outbreak cases, confirming the connection. Further analysis of the accessory genome identified the presence of 1 region unique for 20 of the human outbreak isolates and the 13 confirmed food and veterinary isolates. The identified region had an approximate size of 3,600 bp and contained a ColRNA1-like (92% sequence identity) compatibility gene related to plasmids, 2 genes associated with plasmid regulation (*copG* and *rop*), and 1 hypothetical protein (99% protein similarity with predicted plasmid protein identified in an enteropathogenic *Escherichia coli* 0119:H6 strain [GenBank accession no. AP014807.1]). The region was located on an entire single contig with a higher average read coverage and ≈10% lower GC-content than the average of the genome. The nearest neighbor isolates considered for inclusion in the outbreak did not harbor the plasmid-related region and were not included in the final definition (Table 2).

Our results showed that all 19 isolates defined in 2014 as part of outbreak F clustered with 0 to 3 SNPs differences. Three additional human isolates (all with different MLVA profiles) clustered with 0 SNPs to cases and were included in the new cluster definition. The nearest neighbor isolate was located 20 SNPs away, and the outbreak could clearly be defined. Outbreak and food traceback investigations identified beef as the most likely source. We confirmed 2 samples from companies with 0 to 3 SNPs differences with human cases (Figure 4; Table 2).

Likewise, outbreak G was clearly defined by the SNP analysis. Nine human isolates clustered with 0 to 2 SNPs and separated from nearest neighbor with 31 SNPs. Five isolates previously included were distantly related to the cluster and not included in the new outbreak definition (Figure 4; Table 2). No linked food and veterinary isolates were available; however, we did observe a geographic connection to northern Denmark. Three of 5 excluded patients were interviewed, and no travel to northern Denmark was reported, further indicating the exclusion of the patients from the outbreak.

Last, all 5 isolates previously defined in outbreak H clustered with 0 SNPs. Consumption of a specific swine product was reported by 2 case-patients, and a food sample from the specific product clustered with 0 SNPs (Figure 4; Table 2).

### Influence of Reference Genome

We examined the influence of the choice of reference genome used in the SNP analysis on the cluster formation. As mentioned previously, we analyzed each ST group and each outbreak separately by using an internal de novo assembled reference genome for each ST group and outbreak. We evaluated the size of core-genome used, percentage of reference genome used, and number of called SNPs (Table 3). Our results showed that using an internal reference genome for ST36 yielded an extra 259 SNPs compared with using the complete ST19 genome 14028S. Using an internal reference for ST19 resulted in 47 fewer SNPs and using an internal reference for ST34 resulted in 2 fewer SNPs compared with using the 14028S genome. No extra SNPs were called within the outbreaks for ST19 and ST36, regardless of reference used or group of isolates analyzed. A few extra SNPs were added when analyzing the outbreaks in ST34 separately with an internal reference. Extra SNP resolution was mostly added on the longer branches and not within tight clusters. We also evaluated including poor-quality genomes in the analysis; inclusion resulted in a considerable loss of data (Table 3).

## Discussion

In this retrospective study, we showed that core-genome SNP analysis could be applied for surveillance of *Salmonella* Typhimurium and its monophasic variants. We were able to recover the 8 previously defined outbreaks based on the SNP analysis, epidemiologic data, and food traceback investigations. With the analysis, we could exclude unrelated human isolates and include related isolates not previously defined in the outbreaks based on MLVA. Furthermore, we were able to link possible sources to the outbreaks and reject previously suspected food and veterinary sources. In 4 out of the 8 outbreaks, we could identify the likely source of the outbreak as related to swine. In 1 outbreak, consumption of beef product was confirmed. The remaining 3 outbreaks were not linked to any known sources. In Denmark, *Salmonella* Typhimurium and its monophasic variants are commonly isolated from swine or pork (*2*), and pork meat is considered the main source of infection as observed and reported in many other countries (*9*,*22*–*24*).

The overall phylogeny of all isolates showed 3 groups of isolates corresponding to the ST. The most commonly isolated ST19 and ST34 isolates clustered together, with ST34 isolates defined in a distinct tight cluster. ST36 isolates were separated from ST19 and ST34 isolates with a long branch indicating a distant relation to ST19 and ST34. The distant relation is further confirmed by ST19 and ST34 belonging to e-BurstGroup 1 (eBG1), whereas ST36 is located in eBG138, having 3 out of 7 alleles identical with eBG1 (*25*).

For isolates with ST19 and ST36, the outbreak clusters were well-delimited and separated, as was the case for 2 outbreaks in the ST34 group. For 3 outbreaks with ST34 isolates, the low diversity of the core-genome complicated clear conclusions based on SNP differences between isolates. With further analysis of accessory genes and information from the outbreak investigation, we could more clearly define the outbreaks. Results from the SNP analysis showed that 20% of the reference genome was discarded when analyzing the entire ST34 group, indicating a large amount of accessory data not being used in the analysis. The close core-genome correlates well with the fact that the ST34 is considered a newly expanding clone (*3*,*10*,*11*,*24*). Additionally, the large variation detected in the accessory genome corresponds well with the findings of Petrovska et al. (*26*), which also revealed a high amount of microevolution within a clonal expansion of ST34 in the United Kingdom.

Our results show that the SNP analysis is a suitable typing method in relation to surveillance of *Salmonella* Typhimurium, with the possible exception of some lineages of the monophasic variants. Before applying the method in real time, parameters like isolates analyzed (e.g., ST and clusters), choice of reference genome, and sequence quality need to be addressed and taken into account when setting up a workflow. The reference genome used and the group of isolates analyzed had some, although mostly minor, effect on the SNPs called. However, in general, the choice of reference genome and the selection of isolates analyzed did not change our outbreak definitions. For ST19 and ST34, an overall reference genome, either ST19 or ST34, could be applied. A few extra SNPs were added within outbreaks when analyzing smaller clusters of isolates or outbreaks with an internal reference genome. However, within the tight ST34 group, the few extra SNPs within outbreaks might help in defining some outbreaks more clearly. We observed the largest differences when analyzing the ST36 isolates with a close ST36 reference genome compared with results using an ST19 reference genome. This analysis identified an extra 259 SNPs, and because the ST36 group is distantly related to ST19 and ST34, we recommend using an ST36 reference genome for this group of isolates. 

The parameter that did affect the outcome considerably was the quality of the genomes used. We excluded 6 genomes out of 372 isolates because of poor quality. Including these in the analysis resulted in ≈29% fewer SNPs; therefore, we recommend quality assessment of the genomes before analysis. Last, SNP analysis does not give a static value easily communicated between institutions. Adding new isolates to the analysis results in new calculations and a new core-genome. Potential output differences might occur when different pipelines are used; however, the 3 SNP pipelines used in this study did not result in any major differences in phylogeny and had no influence on the outbreak investigations. A clear consensus of the workflow, quality criteria, and the bioinformatics tools used would resolve practical issues regarding the method. Alternatively, the widely used gene-by-gene approach is faster in real-time surveillance and more easily comparable between laboratories provided identical schemes are used. However, this approach requires expensive software (unless data are uploaded to public repositories) and agreement on schemes and curation of allele databases.

Studies on other *Salmonella* serovars using SNP analysis for outbreak investigation and detection revealed a variable number of SNP cutoff values for defining outbreak clusters, ranging from 0 to 30 SNPs (*14*,*15*,*17*–*19*,*27*,*28*). Likewise, our study shows that SNP cutoffs for outbreaks vary, even within a single serovar, lineage, or clone, and evaluation from outbreak to outbreak is needed. The nature of the outbreak must be taken into account when defining a single outbreak, given that many parameters are possibly affecting the results (e.g., the genetic makeup of the serovar, routes of infection, source types, and time). The SNP analysis and other WGS methods for typing provide a higher discrimination of isolates in comparison with other conventional typing methods (*12*–*19*) and gives the advantage of many additional analyses. However, new typing approaches also lead to many new questions on how to interpret data and, in a surveillance context, how to define outbreak cases and sources. Despite the high resolution of the new typing methods, detailed and extended information on epidemiology and food traceback are still crucial elements in effective surveillance. This study has not only provided valuable information on the core-genome SNP analysis for surveillance and outbreak and source detection but also has given insight into the phylogenetic relationships between isolates of *Salmonella* Typhimurium and its monophasic variants in Denmark.

Technical Appendix 1 Description of 372 isolates of *Salmonella* Typhimurium and its monophasic variants used in an outbreak investigation of *Salmonella* Typhimurium and its monophasic variants, Denmark.

Technical Appendix 2 Description of sequencing procedures and sequence analysis used in an outbreak investigation of *Salmonella* Typhimurium and its monophasic variants, Denmark.
