# Supplementary material for: Investigation of Outbreaks of Salmonella enterica Serovar Typhimurium and Its Monophasic Variants Using Whole-Genome Sequencing, Denmark
Source: Emerg Infect Dis. 2017 Oct;23(10):1631–9. doi: 10.3201/eid2310.161248 (PMC5621559; doi:10.3201/eid2310.161248)
Supplement: Technical Appendix 2 — Description of sequencing procedures and sequence analysis used in an outbreak investigation of Salmonella Typhimurium and its monophasic variants, Denmark. [file 16-1248-Techapp-s2.pdf]

Article DOI: https://doi.org/10.3201/eid2310.161248

Investigation of Outbreaks of *Salmonella enterica* Serovar Typhimurium and Its Monophasic Variants Using Whole-Genome Sequencing, Denmark

Technical Appendix 2

Strain Collection

For this study, 372 isolates of *Salmonella* Typhimurium and its monophasic variants were selected, including 292 human clinical isolates and 80 food and veterinary isolates (Technical Appendix 1). Human isolates were collected from January 2013 until April 2013 and from June 2014 until October 2014, including 8 outbreaks. Food and veterinary isolates were selected based on connection or possible connection to the outbreaks. Further information of the isolates are listed in Technical Appendix 1 (https://wwwnc.cdc.gov/EID/article/23/10/16-1248-Techapp1.xlsx)

Whole-Genome Sequencing

Pure bacterial cultures were cultivated overnight at 37°C on SSI 5% blood agar plates (SSI Diagnostica, Hillerød, Denmark). Genomic DNA was purified at SSI using Qiagen DNeasy Blood and Tissue Kit (Qiagen, Valencia, USA) according to the kit protocol, and at DTU Food using Invitrogen Easy-DNA Kit (Thermo Fisher Scientific, Waltham, USA). Initial DNA concentration was measured and quantified using the Qubit Fluorometer and dsDNA BR/HR Assay Kit (Thermo Fisher Scientific). Sample and library preparation was performed using the Nextera XT v2 DNA Library Preparation kit (used at SSI) or Nextera XT v3 DNA Library Preparation kit (used at DTU Food) (Illumina, San Diego, USA). Libraries were finally purified by Agencourt AMPpure XP System (Beckman Coulter, Indianapolis, USA), and whole-genomes were sequenced using an Illumina Miseq with paired-end technology (250 basepair reads).

Average read coverage at 22 was set as a preliminary quality assessment. Sequences are available at the European Nucleotide Archive (http://www.ebi.ac.uk/ena) under study accession number PRJEB14853.

Sequence Analysis

Sequence reads were de novo assembled using CLC Genomic Workbench (Qiagen) with default settings and a minimum contig size of 500 bp. Statistics from the genome assembly were used as further quality filtering and N50 values higher than 50.000 were accepted. Sequence types (ST) based on the seven gene MLST scheme for *Salmonella enterica* (*1*) were determined from the de novo assembled genomes using the webtool MLST 1.8 (https://cge.cbs.dtu.dk//services/MLST) (*2*).

Core-genome SNPs were detected using an in-house SNP-pipeline based on GATK and BWA-MEM. The core-genome was defined as positions present in all strains, including intergenic regions and with no phage masking, but filtered from recombination events. Reads were mapped and aligned against the complete genome of *Salmonella* Typhimurium strain 14028S (Accession NC_016856.1) (*3*) or against an internal de novo assembled reference genome using BWA v. 0.7.4 (*4*). Aligned reads were sorted, filtered and duplicate reads removed with Elprep v. 1.02 (5). GATK v. 2.5–2 (*6*) was used to call variants, and called variants were filtered by parsing the variant call files using an in-house python script. A minimum read support of 90% was required to make a variant call. Positions with less than ten times depth of coverage or with ambiguous calls in any genome sequence were excluded. Recombination regions were detected based on the base pattern for position of variants in all isolates, where each base pattern was considered a putative branch in a phylogenetic tree. Segments of identical base patterns were sorted by size and considering the frequency of the base pattern of the segments, the probability of observing a segment of a given or longer length was calculated and Bonferroni corrected. Segments with probability below a threshold of 0.05 were removed, and the frequency of the base pattern was reduced by the length of the segment.

Quality of the sequences analyzed in the SNP-pipeline was evaluated by parsing the discarded SNPs file and sorting discarded SNPs based on low depth, ambiguous calls and gaps. Four human and 2 veterinary isolates were excluded from this study based due to poor quality.

Some clusters were re-calculated in the pipeline with the complete genome of strain 14028S or a closely related de novo assembled reference genome to evaluate the potential influence of reference genome on the SNP analysis and to evaluate the influence of analyzing clusters separately. Size of the core-genome and coverage of the reference genome used for SNP analysis was extracted from the pipeline output binary alignment/map files using Samtools v. 0.1.19 (*7*) and an in-house python script. The de novo assembled genomes used as close reference genomes in the SNP analysis were remapped against itself in the SNP pipeline, to check for false positive SNPs.

A selected subgroup of isolates was additionally analyzed using the SNP pipelines NASP (http://tgennorth.github.io/NASP) (*8*) and CSI-phylogeny (https://cge.cbs.dtu.dk/services/CSIPhylogeny) (*9*). Output data with missing SNPs (SNPs called but not present in all genomes) from the NASP pipeline were furthermore used for identification of unique regions in a selected cluster within the ST34 subgroup. Unique regions were BLAST searched against remaining isolates in the cluster using CLC Genomic Workbench. Annotation of the regions was done using PROKKA (*10*), and BLAST search in the NCBI database (https://blast.ncbi.nlm.nih.gov/Blast.cgi) were performed for closer identification of the regions. Possible plasmid regions were identified from de novo assembled genomes using PlasmidFinder 1.3 (https://cge.cbs.dtu.dk//services/PlasmidFinder) (*11*).

Construction of phylogeny of the isolates by multiple alignment of SNPs and calculation of maximum-parsimony (MP) trees were performed using Bionumerics 7.6 (Applied Maths, Sint-Martens-Latem, Belgium).

Outbreaks as Defined by the SNP Analysis

Distribution of the outbreaks A–H and sporadic isolates over time (Technical Appendix 2 Figure). Larger peaks in sporadic isolates were seen, especially in the 2014 period. The peaks were a result of summer season peak in general, and not due to accumulation of e.g., travel related isolates.

References

1. Kidgell C, Reichard U, Wain J, Linz B, Torpdahl M, Dougan G, et al. *Salmonella* typhi, the causative agent of typhoid fever, is approximately 50,000 years old. Infect Genet Evol. 2002;2:39–45. http://dx.doi.org/10.1016/S1567-1348(02)00089-8

2. Larsen MV, Cosentino S, Rasmussen S, Friis C, Hasman H, Marvig RL, et al. Multilocus sequence typing of total-genome-sequenced bacteria. J Clin Microbiol. 2012;50:1355–61. http://dx.doi.org/10.1128/JCM.06094-11

3. Jarvik T, Smillie C, Groisman EA, Ochman H. Short-term signatures of evolutionary change in the *Salmonella enterica* serovar typhimurium 14028 genome. J Bacteriol. 2010;192:560–7. http://dx.doi.org/10.1128/JB.01233-09

4. Li H, Durbin R. Fast and accurate long-read alignment with Burrows-Wheeler transform. Bioinformatics. 2010;26:589–95. http://dx.doi.org/10.1093/bioinformatics/btp698

5. Herzeel C, Costanza P, Decap D, Fostier J, Reumers J. ElPrep: High-performance preparation of sequence alignment/map files for variant calling. PLoS One. 2015;10:e0132868. http://dx.doi.org/10.1371/journal.pone.0132868

6. McKenna A, Hanna M, Banks E, Sivachenko A, Cibulskis K, Kernytsky A, et al. The Genome Analysis Toolkit: a MapReduce framework for analyzing next-generation DNA sequencing data. Genome Res. 2010;20:1297–303. http://dx.doi.org/10.1101/gr.107524.110

7. Li H, Handsaker B, Wysoker A, Fennell T, Ruan J, Homer N, et al.; 1000 Genome Project Data Processing Subgroup. The Sequence Alignment/Map format and SAMtools. Bioinformatics. 2009;25:2078–9. http://dx.doi.org/10.1093/bioinformatics/btp352

8. Lemmer D, Travis J, Smith D, Sahl J. Northern Arizona SNP Pipeline [cited 2016 Jul 1]. http://tgennorth.github.io/NASP

9. Kaas RS, Leekitcharoenphon P, Aarestrup FM, Lund O. Solving the problem of comparing whole bacterial genomes across different sequencing platforms. PLoS One. 2014;9:e104984. http://dx.doi.org/10.1371/journal.pone.0104984

10. Seemann T. Prokka: rapid prokaryotic genome annotation. Bioinformatics. 2014;30:2068–9. http://dx.doi.org/10.1093/bioinformatics/btu153

11. Carattoli A, Zankari E, García-Fernández A, Voldby Larsen M, Lund O, Villa L, et al. In silico detection and typing of plasmids using PlasmidFinder and plasmid multilocus sequence typing. Antimicrob Agents Chemother. 2014;58:3895–903. http://dx.doi.org/10.1128/AAC.02412-14

Technical Appendix 2 Table. Number of isolates selected and sequenced in this study, grouped according to source type, outbreak association and serovar variant

	No. of sequenced isolates			
Source	Total	Linked to outbreak*	Typhimurium	Monophasic variants
Human (2013)	106	54	76	30
Human (2014)	186	68	70	116
Swine	64	14	21	43
Animal	3			
Food/fresh meat	58			
Environmental	3			
Poultry	10	0	1	9
Animal	1			
Food/fresh meat	4			
Environmental	5			
Cattle	3	2	0	3
Food/fresh meat	3			
Feed	3	0	0	3

*Outbreak linked isolates as previously defined based on MLVA, antibiotic susceptibility testing and outbreak/food trace back investigations. Food and veterinary isolates are only marked as linked to outbreaks if the isolates were identified as sources or possible sources.

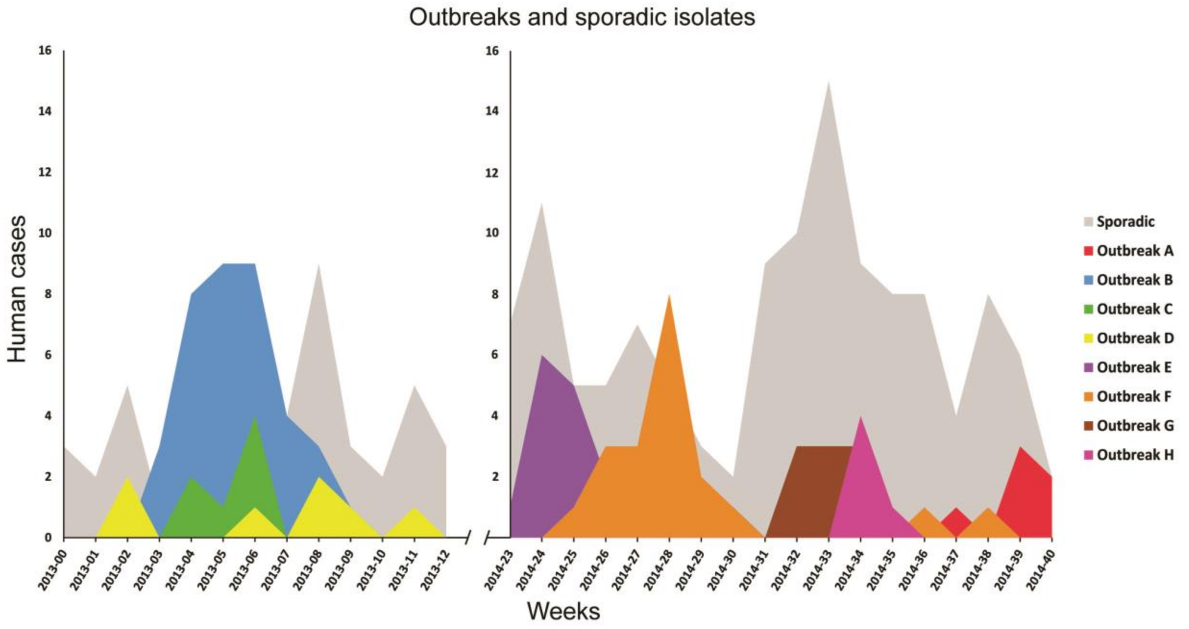

Technical Appendix 2 Figure. Distribution of 8 outbreaks (A–H) and sporadic isolates of *Salmonella* Typhimurium and its monophasic variants over time in weeks. The 8 outbreaks are defined based on core-genome SNP analysis.
